# Renal Malacoplakia Following Obstetric Intervention: A Rare Cause of Acute Kidney Injury in a Young Woman

**DOI:** 10.3390/clinpract15080143

**Published:** 2025-08-03

**Authors:** Letícia Miyuki Ito, Juliana Miki Oguma, André Kiyoshi Miyahara, Marco Aurélio Sales da Veiga, Leandro Favaro, David Wesley de Godoy, Bárbara Antunes Bruno da Silva, Luiz Antônio Moura, Marcelino de Souza Durão, Érika Bevilaqua Rangel

**Affiliations:** 1 Paulista School of Medicina, Federal University of São Paulo, São Paulo 04038-031, SP, Brazil; leticia.miyuki@unifesp.br (L.M.I.); juliana.oguma@unifesp.br (J.M.O.); andre.kiyoshi@unifesp.br (A.K.M.); 2 Department of Medicine, Nephrology Division, Federal University of São Paulo, São Paulo 04038-031, SP, Brazil; marcosalesveiga@gmail.com (M.A.S.d.V.); leandrofavaro01@gmail.com (L.F.); david.weslei.g@gmail.com (D.W.d.G.); barbaraabruno@gmail.com (B.A.B.d.S.); luiz.moura@hrim.com.br (L.A.M.); marcelino.durao@unifesp.br (M.d.S.D.); 3 Instituto Israelita de Ensino e Pesquisa Albert Einstein, Hospital Israelita Albert Einstein, São Paulo 05652-900, SP, Brazil

**Keywords:** urinary tract infection, acute kidney injury, pregnancy, renal malacoplakia

## Abstract

**Introduction**: Renal malacoplakia is a rare chronic granulomatous disease, often associated with immunosuppression and persistent Gram-negative infections, particularly *Escherichia coli*. **Case Presentation**: We present a case involving a 31-year-old woman with hypertension, gestational diabetes, and prior uterine curettage after labor induction for preeclampsia at 23 weeks. She developed urinary sepsis post-procedure. Imaging revealed bilateral nephromegaly, while laboratory tests showed acute kidney injury (KDIGO stage III), anemia, and thrombocytopenia. Blood and urine cultures grew *Escherichia coli*. Renal biopsy confirmed malacoplakia, demonstrating PAS-positive Michaelis–Gutmann bodies and Von Hansemann cells. The patient responded to prolonged antibiotic therapy and supportive care. **Discussion and Conclusion**: This case highlights the importance of considering renal malacoplakia in patients with atypical urinary tract infections and nephromegaly, particularly in obstetric settings. Histopathological confirmation is essential, and timely treatment with intracellularly active antibiotics can lead to favorable outcomes. Early diagnosis is critical to prevent irreversible renal damage.

## 1. Introduction

Acute kidney injury (AKI) is broadly categorized into three etiological groups: pre-renal (due to decreased renal perfusion), intrinsic or renal (due to parenchymal injury), and post-renal (due to urinary tract obstruction) [1]. Pre-renal AKI results from renal hypoperfusion, commonly due to volume depletion, decreased cardiac output, systemic vasodilation, or altered renal vasoregulation (e.g., from medications, hypercalcemia, hepatorenal syndrome, or abdominal compartment syndrome). Intrinsic AKI encompasses a wide range of causes, including vascular and microvascular injuries (e.g., thrombotic microangiopathies such as typical and atypical hemolytic uremic syndrome, disseminated intravascular coagulation, malignant hypertension, scleroderma renal crisis, preeclampsia/HELLP [hemolysis, elevated liver enzymes, low platelet count] syndrome, drug-induced vascular injury, and cholesterol emboli), glomerular diseases, and tubulointerstitial disorders. The latter include acute interstitial nephritis (drug-induced, infectious, or lymphoproliferative), pigment nephropathy, crystal nephropathy, myeloma cast nephropathy, and acute tubular necrosis. Post-renal AKI arises from urinary outflow obstruction and may involve the bladder outlet (e.g., benign prostatic hyperplasia, malignancy, urethral strictures, or blood clots), ureters (bilateral obstruction or unilateral obstruction in a solitary kidney, commonly due to stones, tumors, or retroperitoneal fibrosis), or renal pelvis (e.g., papillary necrosis or obstructing calculi) [1].

Acute pyelonephritis, a tubulointerstitial cause of intrinsic AKI, can lead to renal dysfunction in up to 60% of cases. Risk factors include age > 65 years, complicated or bilateral infections, and initial presentation with septic shock [2].

The differential diagnosis of acute pyelonephritis includes acute interstitial nephritis, light chain cast nephropathy, renal abscess, pyonephrosis, emphysematous pyelonephritis, renal infarction, and nephrolithiasis. Diagnosis is guided by clinical presentation, laboratory findings, and imaging [3].

Chronic pyelonephritis, a long-term consequence of recurrent or inadequately treated acute infections, is often related to structural abnormalities in pediatric populations. In adults, common etiologies include reflux nephropathy, xanthogranulomatous pyelonephritis, and malacoplakia [4].

The term malacoplakia derives from the Greek words *malakos* (soft) and *plakos* (plaque). Malacoplakia is a rare, chronic granulomatous disease, a multisystemic condition most commonly affecting the urogenital tract in immunocompromised individuals. However, it can affect multiple organs, including the gastrointestinal tract, lungs, bones, lymph nodes, and skin. The urinary tract is involved in nearly 60% of cases, with the bladder affected in 40%, the ureters in 11%, and the renal pelvis in 10% of the cases [5]. Its pathophysiology involves defective lysosomal degradation in macrophages, resulting in cytoplasmic accumulation of bacterial debris and the formation of Michaelis–Gutmann bodies and Von Hansemann cells [5,6,7,8,9]. Malacoplakia is frequently associated with persistent Gram-negative bacterial infections, particularly *Escherichia coli* [5,7,8,10].

This report describes a rare case of renal malacoplakia in a female patient with a history of preeclampsia, gestational diabetes, and recurrent urinary tract infections. She underwent labor induction in the second trimester, followed by uterine curettage. Subsequently, she developed AKI, prompting consultation with the Nephrology team. We aimed to correlate the clinical, laboratory, radiological, and histopathological findings with the progression of kidney function.

## 2. Case Presentation

The study was conducted in accordance with the Declaration of Helsinki and approved by the Ethics and Research Committee of the Federal University of São Paulo, São Paulo, Brazil (number CAAE: 85557524.9.0000.5505; date of approval: 16 April 2025).

A 31-year-old White woman with a medical history of systemic arterial hypertension (15 years, treated with losartan, amlodipine, and atenolol), grade I obesity, a 12 pack-year smoking history, and recurrent urinary tract infections, presented during her sixth pregnancy (at 23 weeks’ gestation) with gestational diabetes mellitus (treated with dietary adjustments and NPH insulin with pre-natal fasting blood glucose of 92 mg/dL). Her obstetric history included five previous pregnancies, the most recent complicated by preeclampsia, resulting in premature labor at 35 weeks’ gestation, and a miscarriage requiring uterine curettage five years prior. Her family history was significant for maternal hypertension, diabetes mellitus, and chronic kidney disease requiring hemodialysis.

She was hospitalized at Hospital São Paulo (São Paulo, SP, Brazil) for preeclampsia, presenting with hypertensive crises, headache, epigastric pain, vomiting, and papilledema on fundoscopy. At admission, she was receiving amlodipine (10 mg/day), hydralazine (200 mg/day), and methyldopa (2000 mg/day), with a blood pressure of 151/95 mmHg. Initial laboratory results showed hemoglobin of 9.8 g/dl, leukocyte count of 7540/µL, platelet count of 125,000/mm^3^, serum creatinine of 0.96 mg/dL, fasting blood glucose of 85 mg/dL, lactate dehydrogenase (LDH) of 230 U/L (reference: <250 U/L), aspartate aminotransferase (AST) of 32 U/L (reference: <32 U/L), and alanine aminotransferase (ALT) of 48 U/L (reference: <33 U/L). Urinalysis revealed proteinuria of 1.5 g/L, leukocyturia of 9240/mL, erythrocyturia of 3520/mL, and negative nitrite. Subsequent testing showed worsening renal function, with creatinine rising to 1.24 mg/dL, LDH increasing to 308 U/L, AST to 37 U/L, and ALT to 51 U/L. Urinalysis at that time revealed significant proteinuria (>5 g/L), marked leukocyturia (27,270/mL), and erythrocyturia (102,800/mL), but with negative nitrite, and a urinary albumin-to-creatinine ratio of 6320 mg/g. Therefore, preeclampsia was considered a potential diagnosis [11,12]. Obstetric evaluation revealed intrauterine growth restriction (fetal weight below the 10th percentile for gestational age) and an abnormal umbilical artery to middle cerebral resistance index ratio (>1), indicating fetal circulatory compromise. Labor was induced, followed by uterine curettage. The female neonate weighed 495 g and had Apgar scores of 2, 4, and 8 at 1, 5, and 10 min, respectively. She died 24 h after birth. The patient was discharged three days later. However, one day after discharge, she developed bilateral flank pain, fever (40 °C), dysuria, urinary urgency, and malodorous urine.

Three days after symptom onset, the patient sought medical attention. At admission —6 days following uterine curettage—she appeared pale (2+/4+), dehydrated (1+/4+), afebrile (36 °C), with a blood pressure of 98/63 mmHg and a heart rate of 105 bpm. Abdominal examination revealed deep bilateral flank tenderness, more intense on the right side, without evidence of peritoneal irritation or palpable masses. Neurological, cardiovascular, and respiratory examinations were unremarkable.

A presumptive diagnosis of acute pyelonephritis was made, and empirical antibiotic therapy with ceftriaxone was initiated. Urine and blood cultures confirmed *Escherichia coli* infection. Laboratory investigations revealed non-oliguric acute kidney injury (AKI), KDIGO stage 3 (baseline creatinine 0.96 mg/dL), thrombocytopenia, and anemia (Table 1). Serologies for hepatitis B, hepatitis C, HIV, and syphilis were negative. Serum complement levels were within normal limits. Urinalysis showed 60 leukocytes/field, 15 erythrocytes/field, and proteinuria of 1.84 g/dl. The urinary albumin-to-creatinine ratio was 1220 mg/g. Initial renal ultrasound revealed no abnormalities, with no signs of urinary tract obstruction (Table 1). Fundoscopy revealed chronic papilledema secondary to hypertensive retinopathy.

**Figure 1:**
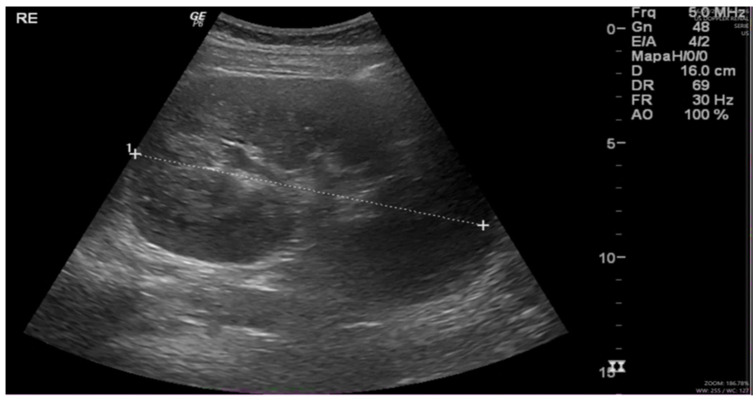
Renal ultrasound: kidneys in orthotopic position, with preserved contours and increased dimensions. Renal parenchyma with homogeneous thickness and echogenicity. Right kidney: 15.8 cm; parenchymal thickness: 2.2 cm. Left kidney: 16.0 cm; parenchymal thickness: 2.0 cm. No evidence of pelvicalyceal system dilation. Minimal amount of free fluid in the pelvis. (“1 to +” refers to the measurement of the left kidney’s size).

**Figure 2:**
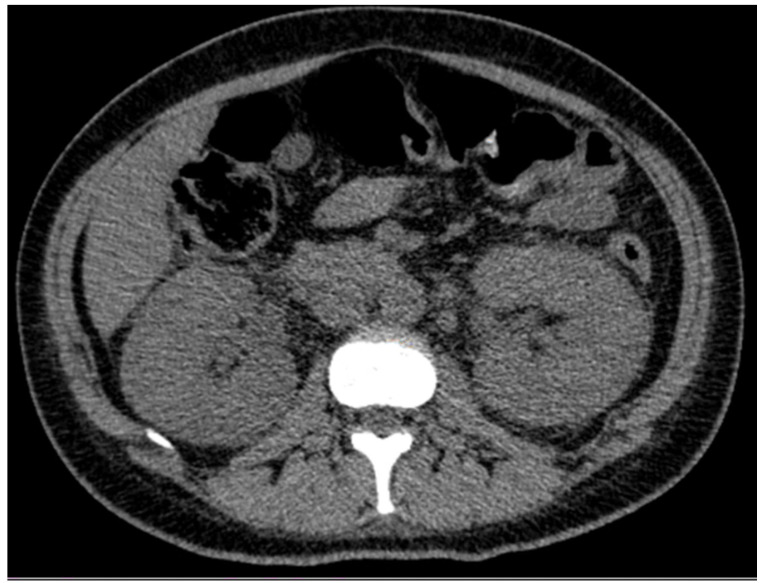
Abdominal and pelvic computed tomography scan revealing bilateral renal enlargement.

Following nearly three weeks of treatment with ceftriaxone, the patient exhibited partial recovery of kidney function, with creatinine decreasing from 4.43 to 1.81 mg/dL (Table 1). A follow-up ultrasound was performed, revealing an increase in kidney size (Figure 1) compared to the initial examination. Bladder presented with good filling, regular walls, and anechoic content. These findings were confirmed by abdominal computed tomography (CT), which demonstrated bilateral nephromegaly (Figure 2).

**Figure 3:**
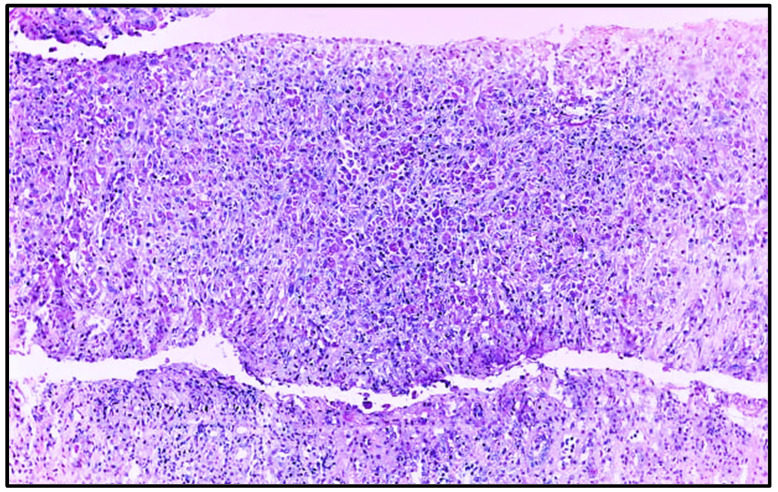
Renal biopsy histopathology, showing extensive inflammatory infiltrate and absence of glomeruli and tubules in the sample (Hematoxylin–eosin, original magnification, ×100).

**Figure 4:**
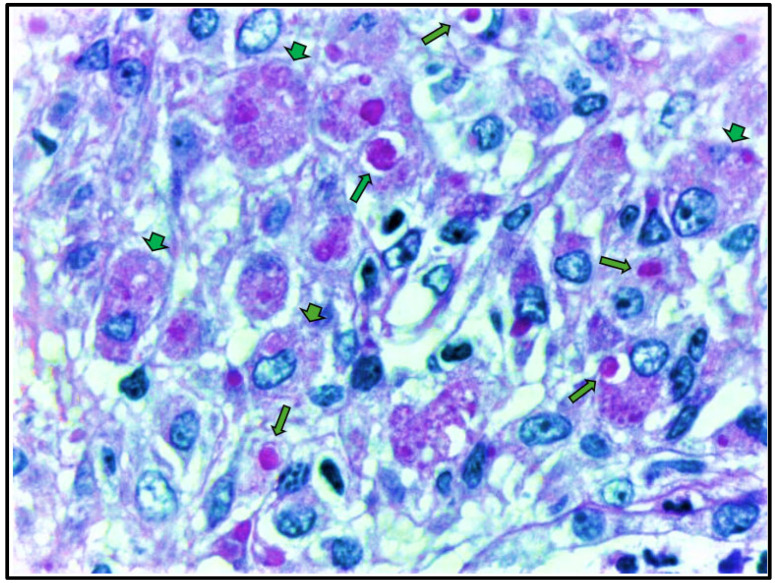
Renal biopsy histopathology demonstrates large histiocytes with foamy and granular cytoplasm (von Hansemann cells), indicated by arrowheads, and PAS-positive granules characteristic of Michaelis–Gutmann bodies, indicated by arrows. (Periodic acid–Schiff stain, original magnification, ×100).

To further investigate the partial recovery of kidney function, nephromegaly, and elevated C-protein levels (Table 1), a renal biopsy was performed. Renal biopsy (Figure 3 and Figure 4) showed cortical and medullary parenchyma infiltrated by polygonal cells with abundant PAS-positive granular cytoplasm, arranged in solid epithelioid patterns along with small aggregates of neutrophils and plasma cells. One glomerulus exhibited retracted capillary loops with preserved cellularity. Direct immunofluorescence revealed granular IgM (+) and C3c (++) deposits; other immunoglobulins, complement components, and fibrinogen were negative. These findings supported a diagnosis of malacoplakia, with ischemic glomerular retraction and diffuse cellular infiltration. The predominance of C3 suggested an infection etiology.

The patient developed urinary sepsis, which resolved following a 21-day course of ceftriaxone (1 g every 12 h), followed by intravenous ciprofloxacin (400 mg every 12 h for six days). Shortly after initiating ciprofloxacin, she developed severe leukopenia (total leukocyte count: 1330/µL), with marked neutropenia (neutrophils: 126/µL, 9.5%) and relative lymphocytosis (lymphocytes: 871/µL, 65%) (Table 1). She was treated with granulocyte colony-stimulating factor (G-CSF; filgrastim, Granulokine, Amgen Manufacturing Limited, Juncos, Puerto Rico), which was discontinued upon the development of leukocytosis.

The patient requested early discharge and was scheduled for outpatient follow-up with the nephrology team. She was instructed to continue ciprofloxacin to complete a four-week course of antibiotic therapy. Additionally, she was diagnosed with anemia of inflammation and initiated erythropoietin therapy (4000 IU three times per week).

She remained dialysis-independent and showed significant clinical improvement, with resolution of thrombocytopenia and partial renal recovery (serum creatinine: 1.46 mg/dL; estimated glomerular filtration rate [eGFR]: 49 mL/min/1.73 m^2^). At that time, her medications included losartan (100 mg/day), amlodipine (10 mg/day), atenolol (100 mg/day), and hydrochlorothiazide (25 mg/day). Glycemic control was within normal limits, with a fasting blood glucose of 85 mg/dL and a glycated hemoglobin of 4.9%, and she no longer required insulin therapy. However, the patient was subsequently lost to follow-up 13 months after the onset of symptoms.

## 3. Discussion

In this study, we report a case of pregnancy-related AKI secondary to bilateral renal malacoplakia with a positive urine culture for *Escherichia coli* in a young patient with gestational diabetes, recurrent urinary tract infection, preeclampsia treated with anti-hypertensive drugs, and pregnancy interruption followed by uterine curettage, and who showed partial recovery of kidney function following antibiotic therapy.

Pregnancy-related acute kidney injury (PR-AKI) is historically considered rare, with an estimated incidence of fewer than 1 in 20,000 pregnancies. However, this rate is increasing globally, primarily due to the rising prevalence of associated risk factors [12]. PR-AKI contributes substantially to maternal morbidity and mortality, with reported maternal mortality rates ranging from 30% to 60%. It is also linked to long-term complications, including chronic hypertension, chronic kidney disease (CKD), and cardiovascular disease [12]. The etiologies of PR-AKI are typically categorized as (a) pre-renal causes, including hemorrhage, hypovolemia (e.g., due to hyperemesis gravidarum), sepsis, and congestive heart failure; (b) intrinsic renal causes, such as acute tubular necrosis, renal cortical necrosis, thrombotic microangiopathies, preeclampsia/HELLP syndrome, acute fatty liver of pregnancy, glomerulonephritis, and acute interstitial nephritis; and c) post-renal causes, which involve mechanical obstruction due to postsurgical complications, malignancies, or ureteropelvic obstruction from the gravid uterus. Pre-renal causes are more commonly observed in the first trimester, while intrinsic and post-renal causes typically manifest later in pregnancy [11,12].

In the postpartum period, AKI (PP-AKI) is relatively uncommon, with a reported incidence ranging from 0.81% to 4.5% [12,13,14,15,16]. Identified risk factors include both antenatal and intrapartum complications, such as preeclampsia, prolonged rupture of membranes, emergency cesarean section, and excessive blood loss [15]. In certain cohorts, the primary causes of early AKI in obstetric patients include infection (17–48%), preeclampsia (26–28.3%), hemorrhage (20.8–25%), and unknown etiologies (~15%) [16,17]. While most patients (~75–80%) experience full renal recovery, approximately 10–20% may not regain baseline kidney function [16,17,18], consistent with our findings.

In our case, the initial diagnosis was acute pyelonephritis, based on clinical presentation and laboratory findings, in the setting of preeclampsia, gestational diabetes, and a history of recurrent urinary tract infections, with no evidence of peri- or postpartum hemorrhage nor any signs post-renal obstruction. However, due to the absence of renal function recovery after three weeks of appropriate antibiotic therapy, a repeat ultrasound of the urinary tract was performed, revealing bilateral nephromegaly. Consequently, a subsequent renal biopsy, considered the diagnostic gold standard, confirmed the diagnosis of renal malacoplakia.

Malakoplakia typically affects immunosuppressed or transplant patients, as well as individuals with comorbidities such as diabetes, AIDS, and alcoholism [19,20]. To the best of our knowledge, this case report presents a unique and meticulously documented instance of renal malacoplakia in a young postpartum patient receiving obstetric care—a clinical context not previously well described in the literature, as shown in Table 2 [13,21,22,23,24,25,26,27,28,29,30,31,32,33,34,35,36,37]. In this review, we present demographic data, key laboratory findings, treatment approaches, and clinical outcomes in patients with malacoplakia affecting native and transplanted kidneys.

In this case, the temporal association between uterine curettage and the onset of clinical symptoms and laboratory abnormalities—occurring within six days—raises the possibility of a causal relationship. The most common postpartum infections include surgical site infections and endometritis, while asymptomatic bacteriuria has been documented in 8–12% of postpartum women. However, only approximately 25% of these women will develop dysuria or other symptoms of urinary tract infection [38].

Uterine curettage may be associated with urinary tract infection through several mechanisms, including disruption of the normal vaginal and periurethral flora, facilitating the ascent of uropathogens into the bladder or upper urinary tract; urinary catheterization during the procedure; ascending infection due to suboptimal sterility; iatrogenic contamination; and retained products of conception or incomplete uterine evacuation, which can serve as a nidus for infection and lead to secondary urinary tract involvement through hematogenous spread or direct extension [38,39].

Notably, this patient had a history of recurrent urinary tract infections, suggesting an underlying predisposition that had not been previously identified. In combination with a surgical intervention, a compromised renal microenvironment due to hyperglycemia from gestational diabetes and AKI by preeclampsia, these factors likely contributed to the development of a refractory and chronic pyelonephritis.

In this case, gestational diabetes may have contributed to urinary infection and AKI, as diabetes is a recognized risk factor for AKI [40]. In the pregnancy setting, in a cohort study involving approximately 500 women with and without diabetes or gestational diabetes mellitus, the frequencies of asymptomatic bacteriuria at 12 weeks (4.7% vs. 2.3%) and urinary tract infection (16.8% vs. 12.9%) were comparable [41]. However, a recent meta-analysis demonstrated a statistically significant association between gestational diabetes and urinary tract infection, with a pooled odds ratio (OR) of 1.3 [42]. This association may be attributed to glycosuria and the anatomical and physiological changes of pregnancy, which predispose to bacterial colonization and infection.

Renal involvement in malakoplakia is often bilateral and can result in AKI [5,21,43]. The disease predominantly affects adults over the age of 40 years and is two [8] to four [5] times more common in women. *Escherichia coli* is the most common pathogen [5,10,21], as also shown in Table 2. Clinical features are nonspecific and may include fever, flank or lumbar pain, and palpable masses [21,24,43,44], which were also found in the present case.

Additional investigation of malacoplakia includes imaging analyses. These findings vary, ranging from normal renal morphology to nephromegaly with functional impairment [7,8,21,22,24,43]. Renal malacoplakia may present as unifocal or more frequently multifocal patterns (~75%), with bilateral involvement occurring in 50% of cases [5]. Differential diagnoses include renal abscess, granuloma (e.g., tuberculosis), xanthogranulomatous pyelonephritis, lymphoma, and metastatic disease [5,7,8,25]. Clinical features such as fever, flank pain, and palpable masses are nonspecific and overlap with other renal pathologies [7,8,23].

In cases of renal abscess, the clinical presentation typically includes abrupt onset of symptoms, febrile urinary tract infection, flank pain, and leukocytosis. Imaging studies reveal hypodense, fluid-filled lesions with rim enhancement; the presence of gas within a cystic or low-attenuation mass is highly suggestive of abscess formation [45]. Emphysematous pyelonephritis, a life-threatening infection of the renal parenchyma, is characterized by the presence of gas within the renal parenchyma and the retroperitoneal space [45]. In contrast, granulomatous disease is often characterized by fever, weight loss, hematuria, sterile pyuria, and systemic manifestations. Imaging findings include calcifications, cortical scarring, and cavitary lesions [46]. Xanthogranulomatous pyelonephritis (XGP) presents in the context of chronic urinary tract obstruction, frequently associated with staghorn or ureteral calculi and recurrent infections. Imaging typically shows an enlarged kidney, with multiple low-density, rounded areas corresponding to dilated calyces or inflammatory infiltrates, along with loss of corticomedullary differentiation [47]. Renal lymphoma, most commonly observed in immunocompromised patients or as part of disseminated systemic disease, rarely occurs as a primary renal neoplasm. It usually presents with systemic symptoms such as fever and weight loss. Imaging reveals bilateral or multifocal homogeneous or hypodense renal masses, sometimes with invasion from adjacent retroperitoneal or perirenal lymphomatous involvement [48]. Renal metastases most frequently originate from primary malignancies of the lung, colon, prostate, or thyroid. Imaging features include multiple, bilateral, hypodense or isodense lesions, often wedge-shaped and located near the renal capsule, with limited exophytic growth. These contrast with primary renal tumors, which tend to be solitary, unilateral, non-wedge-shaped masses that demonstrate an exophytic growth pattern with early capsular invasion [49].

Therefore, the diagnosis of malacoplakia should include a histopathological investigation. Its diagnosis is characterized by the presence of Michaelis–Gutmann bodies and Von Hansemann cells, which are large macrophages with abundant and eosinophilic cytoplasm in H&E staining, whereas in PAS, they stain strongly positive with a foamy and granular cytoplasm due to the accumulation of lysosomal debris containing glycoprotein and carbohydrates derived from partially digested bacteria. These findings highlight the presence of macrophage dysfunction that results in impaired lysosomal degradation and cytoplasmic accumulation of bacterial remnants. In PAS, the Michaelis–Gutmann bodies are round inclusions found within or outside the cytoplasm and are considered pathognomonic for malakoplakia [5,6,7,8,9], as also documented in Table 2.

Histopathological findings vary across differential diagnoses of renal masses. In renal abscesses, histology reveals dense neutrophilic infiltration with areas of suppuration [50]. In renal tuberculosis, granulomatous inflammation is observed, often with caseating necrosis [46]. Xanthogranulomatous pyelonephritis is characterized by the presence of foamy macrophages, multinucleated giant cells, and chronic inflammatory infiltrates [4]. In renal lymphoma, a monomorphic lymphoid infiltrate is typically observed, with immunohistochemistry demonstrating CD20-positive B cells [51]. In cases of renal metastasis, atypical epithelial or mesenchymal cells are identified, with the primary origin confirmed by specific immunohistochemical markers [49].

Malacoplakia treatment usually involves prolonged antibiotic therapy with agents that have good intracellular penetration, such as fluoroquinolones [7,8,10,21], trimethoprim [21,44], or azithromycin [21,28], as well as other antibiotics (Table 2). Bethanechol, a cholinergic agonist, may be added to the treatment to increase intracellular 3′-5′ guanosine monophosphate (cGMP) levels in macrophages, thereby preventing the impaired release of lysosomal enzymes required for the digestion of phagocytosed bacteria [21,23,36,52]. Management may also include adjustment of immunosuppressive therapy modulation, and, in some cases, surgical resection [7,8,10,21], as also shown in Table 2. Accurate diagnosis via renal biopsy [53] is critical to avoid unnecessary surgery when conservative treatment is adequate.

This case illustrates the hallmark features of renal malacoplakia, including urinary tract infection symptoms, positive *Escherichia coli* cultures, nephromegaly, AKI, and characteristic radiological and histopathological findings. These manifestations occurred following uterine curettage after premature labor induced by preeclampsia in a young woman with gestational diabetes. This case highlights the importance of integrating clinical, obstetric, and nephrological data, encompassing imaging, laboratory results, and histopathological findings. Early recognition, targeted antibiotic therapy, and timely renal biopsy are critical in managing atypical urinary tract infections in postpartum patients. Despite treatment with prolonged antibiotics and supportive care, only partial renal recovery was achieved. The patient progressed to chronic kidney disease (CKD), evidenced by a persistently reduced eGFR below 60 mL/min/1.73 m^2^, as also demonstrated in Table 2, along with ongoing urinary abnormalities, including albuminuria, leukocyturia, and dysmorphic hematuria. Long-term follow-up in cases of renal malacoplakia is critically important, given the potential risk of CKD progression—an outcome that remains poorly characterized in this clinical context.

## 4. Conclusions

Although rare, renal malacoplakia may be considered in the differential diagnosis of atypical urinary tract infections and renal masses. Histopathological evaluation remains important for establishing a definitive diagnosis. As illustrated by this case, appropriate antibiotic therapy initiated in a timely manner may result in a favorable clinical outcome. This report contributes to the limited but expanding literature on the clinical and morphological features of renal malacoplakia in obstetric patients.

## Figures and Tables

**Table 1:** Laboratory parameters over time.

	Day 1	Day 28	Day 35	Day 48	Day 77	Day 193	Reference Values
Hemoglobin (g/dL)	8.7	7.8	9.0	9.5	13.2	14.1	12–15.5
Leukocytes (/µL)	27,650	1330	55,800	9900	6400	7000	3500–10,500
Platelets (/mm^3^)	80,000	572,000	145,000	212,000	257,000	192,000	150,000–450,000
Creatinine (mg/dL)	4.43	1.81	1.88	1.61	1.50	1.46	0.6–1.0
eGFR (mL/min/1.73 m^2^)	13.1	38.2	36.2	43.3	47.2	49	>90
Urea (mg/dL)	134	46	32	42	42	41	10–45
Potassium (mEq/L)	3.5	4.7	3.5	3.2	3.1	3.3	3.6–5.0
Sodium (mEq/L)	131	135	136	135	136	136	135–145
Glucose (mg/dL)	118	80				90	70–99
pH	7.38		7.31	7.34	7.38	7.38	7.33–7.43
Bicarbonate (mmol/L)	16.8		21.1	29.1	26	26	23–27
C-reactive protein (mg/dL)	428	67	11.5	8.1	1.71		<1.0
Urine (Protein in g/dL; Leukocytes and Erythrocytes *: per mL or per field)	Protein: >0.5 L: >1 × 10^6^/mL; E: 299 × 10^3^/mL	Protein: 0.75 L: 23 × 10^3^/mL; E: 6 × 10^1^/mL			Protein: 1.09 L: 80; E: 40	Protein: 0.60 L: 60; E: 50	Protein: <0.15 L and E: <10.000/mL or L: 10; E: 10 (per field)
Urinary albumin-to-creatinine ratio (mg/g) 24-h Proteinuria (g)	2.17	2220 *(day 12)*			466 1.39	247 0.68	<30 <0.15
Ultrasound (kidney size, in cm)	RK = 12.4 LK = 13.3	RK = 15.8 LK = 16 (*day 25*) + kidney biopsy (*day 27*)					
Antibiotic Ceftriaxone Ciprofloxacin	+ (D1 to 24)	+ (D25)	+	+	+ (D65)		

* All urine samples demonstrated erythrocyte dysmorphism, commonly graded as ++ (range: negative to +++, reference value: negative). eGFR = estimated glomerular filtration rate; L = Leukocytes; E = Erythrocytes. RK = Right Kidney; LK = Left Kidney.

**Table 2:** Review of the literature regarding demographic data, urine culture results, antibiotic and surgical treatment, and outcome.

Number of Patients	Age	Gender	Bacteria Identified	Antibiotic Treatment	Surgery	Outcome	Type of Article	Reference
26	4 wks to 82 yrs	Female (*n* = 20, 77%) Male (*n* = 4, 15.4%) not specified (*n* = 2)	*E. coli* (*n* = 20, 77%) Not identified (*n* = 5) Lactose-fermenting bacilli (*n* = 1)	Ciprofloxacin, Norfloxacin or Levofloxacin (*n* = 11, 42.3%) TMP-SMZ (*n* = 6; 23.1%) Cephalosporin (*n* = 2, 7.7%), Rifampin (*n* = 2), Ceftizoxime, Gentamicin, Ceftriaxone, Cefuroxime (*n* = 1, each), not specified (*n* = 2)	Yes (*n* = 6, 23.1%)	Survival (*n* = 24, 92.3%), death (*n* = 2, 7.7%), HD requirement at 12 m (*n* = 3, 11.5%), Creatinine: 2.33 ± 1.52 mg/dL (median = 2.0 mg/dL)	Case report and systematic review	Tam et al., 2003 [21]
1	56 yrs	Female	Not identified	TMP-SMZ	No	Survival; creatinine 3.15 mg/dL	Case report	Sheerin NS et al., 2003 [22]
1	10 yrs	Male	Not identified	TMP-SMZ	No	Survival	Case report	Kajbafzadeh et al., 2004 [23]
1	37 yrs	Male	Not identified	None	Yes	Survival	Case report	Wilenberg et al., 2004 [24]
1	42 yrs	Female	Not identified	Ceftriaxone	Yes	Survival	Case report	Cury et al., 2007 [25]
1	56 yrs	Female	*E. coli*	Gentamycin, levofloxacin	No	Survival, creatinine 2.54 mg/dL	Case report (kidney transplant)	Augusto et al., 2008 [13]
1	71	Female	*E. coli*	Ciprofloxacin, vancomycin, aztreonam	Yes	Survival, kidney function not reported	Case report	Richter et al., 2011 [26]
1	62 yrs	Female	Not identified	Ciprofloxacin, TMP-SMZ, piperacillin/tazobactam	Yes	Survival, creatinine 3.4 mg/dL	Case report	Purnell et al., 2015 [27]
2	52 yrs 63 yrs	Female Female	*E. coli* *E. coli*	Ciprofloxacin, azithromycin Cefepime, ceftriaxone, ciprofloxacin, azithromycin	Yes (drainage) No	Survival, creatinine clearance 53 mL/min and 50 mL/min	Case reports (kidney transplant)	Kinsella et al., 2021 [28]
1	Her 50s	Female	*E. coli*	Yes, not reported	Yes (liver and diaphragm invasion)	Survival, kidney function not reported	Case report	Grunhut et al., 2022 [29]
20	14–84 yrs	Female	*E. coli* (*n* = 17, 85%), Negative (*n* = 2), not reported (*n* = 1)	Yes, not reported	Not reported	Survival, kidney failure (*n* = 4)	Case report and systematic review (kidney transplant)	Triozzi et al., 2022 [30]
1	63 yrs	Female	*E. coli*	Ceftriaxone, levofloxacin	No	Survival, creatinine 1.3 mg/dL	Case report	Haq et al., 2023 [31]
1	49 yrs	Female	*Klebsiella pneumoniae*	Yes, not reported	No	Survival, creatinine 2.5 mg/dL	Case report (kidney transplant)	Vishwajeet et al., 2023 [32]
1	55 yrs	Male	*E. coli*	Yes, Ceftriaxone, cefdinir, TMP-SMZ, levofloxacin	No	Survival, creatinine 1.5 mg/dL	Case report (kidney transplant)	Rustom et al., 2023 [33]
1	33 yrs	Female	Not identified	Yes, not reported	Yes	Survival, normal function	Images in Clinical Medicine	Bagnasco et al., 2024 [34]
1	53 yrs	Female	Not reported	Yes, not reported	Yes	Not reported	Case report	Daghdagh et al., 2024 [35]
1	32 yrs	Female	*E. coli*	Tigecycline, aztreonam, and minocycline	Yes	Survival, creatinine 1.2 mg/dL (eGFR 61 mL/min/1.73 m^2^)	Case report	Fatola et al., 2024 [36]
2	55 yrs 25 yrs	Female Male	*E. coli* *Enterococcus*	Minocyclin Ceftriaxone and daptomycin, cefpodoxime and doxycycline	Yes (drainage) Yes (nephrostomy)	Survival, Creatinine 2.0 mg/dL and 2.5 mg/dL, respectively	Case reports (kidney transplant)	Abbasi et al., 2025 [37]

Wk = week; yr = year; TMP-SMZ = trimethoprim–sulfamethoxazole; HD = hemodialysis; eGFR = estimated glomerular filtration ratio; m = month.

## Data Availability

The data presented in this study are available upon request from the corresponding authors. The data are not publicly available due to the inclusion of clinical patient information.
